# Protocol for determining gut microbiota metabolites as substrates in mouse metabolism

**DOI:** 10.1016/j.xpro.2025.104325

**Published:** 2026-01-07

**Authors:** Han Fang, Dana Kukje Zada, Nicole G. Barra, Rodrigo Rodrigues e-Lacerda, Jonathan D. Schertzer

**Affiliations:** 1 Department of Biochemistry and Biomedical Sciences, Farncombe Family Digestive Health Research Institute, and Centre for Metabolism, Obesity and Diabetes Research, McMaster University, Hamilton, ON L8N 3Z5, Canada

**Keywords:** metabolism, microbiology, mass spectrometry

## Abstract

Here, we present a protocol for studying how microbiota-derived substrates fuel host mouse metabolism using stable isotope tracing. We describe steps for integrating ^13^C tracing in bacterial culture with ^13^C-labeled CO_2_ tracing in free-moving mice. We then detail procedures for determining the metabolic flux and fate of gut microbiota-derived substrates in the host through comparison between germ-free mice and monocolonized or specific pathogen-free mice. This protocol provides a framework for determining metabolic interactions between the gut microbiota and host.

For complete details on the use and execution of this protocol, please refer to Fang et al.^1^

## Highlights

• Steps for setting up a stable isotope gas analyzer in free-moving mice
• Instructions for substrate administration and ^13^CO_2_ measurement in expired breath
• Guidance on data analysis integrating ^13^C tracing in culture and mice

## Abstract (editor highlights)

Publisher’s note: Undertaking any experimental protocol requires adherence to local institutional guidelines for laboratory safety and ethics.

## Graphical abstract

**Figure:**
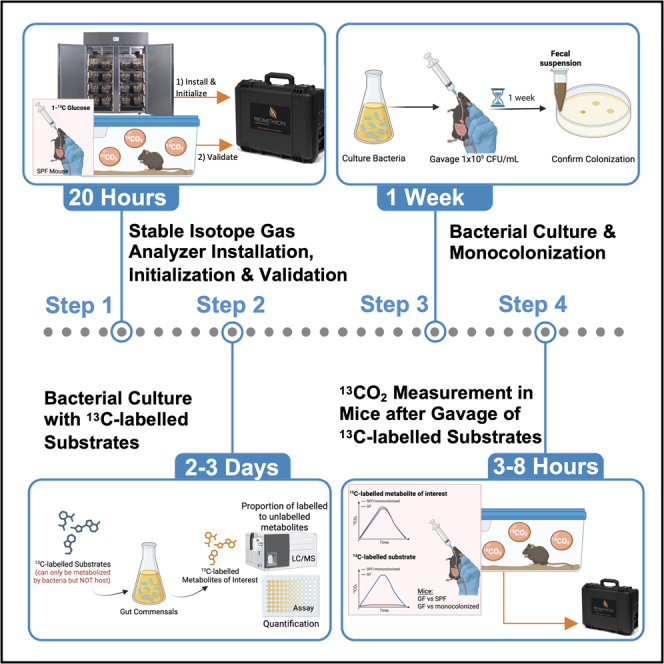

## Before you begin

Gut microbiota produces various metabolites that can serve as substrates for host metabolic pathways.^2^ As an example, both bacteria and host tissues can produce either enantiomer of lactate. L-lactate is mainly produced in the host, primarily in the muscle, which fuels the Cori cycle. We found that bacteria produce most of the D-lactate that is circulating in mice. We determined whether D-lactate originating from gut bacteria can also serve as a fuel source for host glucose metabolism via an alternate branch in the Cori cycle. Although methods existed to track the fate of stable isotope-labeled exogenous substrates, we devised a method to track endogenously produced metabolites derived from the gut microbiota, which then can be used by the host.

In this protocol, we measured a stable isotope of carbon in exhaled breath of mice after “feeding” bacteria in the gut using oral gavage in order to reveal the oxidation of labeled substrates.^3^ We first measured ^13^C-labeled lactulose in bacterial culture, where it is metabolized by commensals to produce ^13^C-labeled D-lactate. Next, we orally administered ^13^C-labeled lactulose, which cannot be processed by the host, to specific pathogen-free (SPF) mice, monocolonized mice, and germ-free (GF) mice to monitor ^13^CO_2_ production in free moving mice. This two-step strategy provides evidence that microbial-derived D-lactate can be utilized by the host as a fuel source for glucose metabolism.

Although this protocol is exemplified using microbial-derived D-lactate, it can be readily adapted to other microbial-derived metabolites, provided that selected substates are exclusively metabolized by microbial enzymes and not by host pathways. Furthermore, this protocol is compatible with the use of ^18^O-labeled substrates.

### Innovation

The innovation of this protocol lies in its integrated cross-system isotope tracing design, which links microbial metabolite production with host utilization in real time. By combining ^13^C-labeling in bacterial cultures with *in vivo* ^13^CO_2_ tracing in SPF, monocolonized and GF mice, this method establishes direct link between microbial metabolite production and host substrate utilization. This protocol provides evidence that specific microbiota-derived metabolites, such as D-lactate from *Lactobacillus* species, enter host metabolic pathways and are oxidized for energy.

### Institutional permissions

All animal experiments were done in accordance with the Canadian Council on Animal Care and approved by the Animal Review Ethics Board (AREB) at McMaster University.

Researchers using this protocol must have permission and follow the rules of their authorities on animal care and usage.

### Installing the stable isotope gas analyzer equipment

**Timing: 30 min**

**Figure 1:**
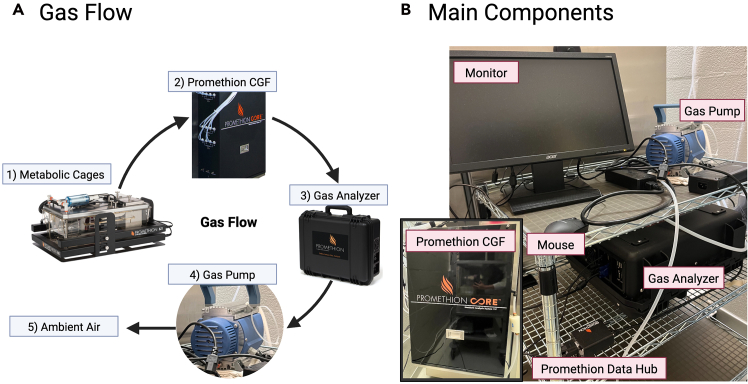
Stable Isotope Gas Analyzer components (A) Gas flow from the metabolic cages to the gas analyzer. (B) Main components of ^13^CO_2_ measurements.

This protocol describes detailed instructions for installing the Sable Isotope Gas Analyzer in conjunction with the Promethion Core Metabolic and Behavioral Measurement System. The direction of gas flow was shown in Figure 1A.

1. Place the stable isotope gas analyzer, gas pump, and monitor on a cart provided (Figure 1B).

***Note:*** The stable isotope gas analyzer should be placed on a stable surface and lifted.

**Figure 2:**
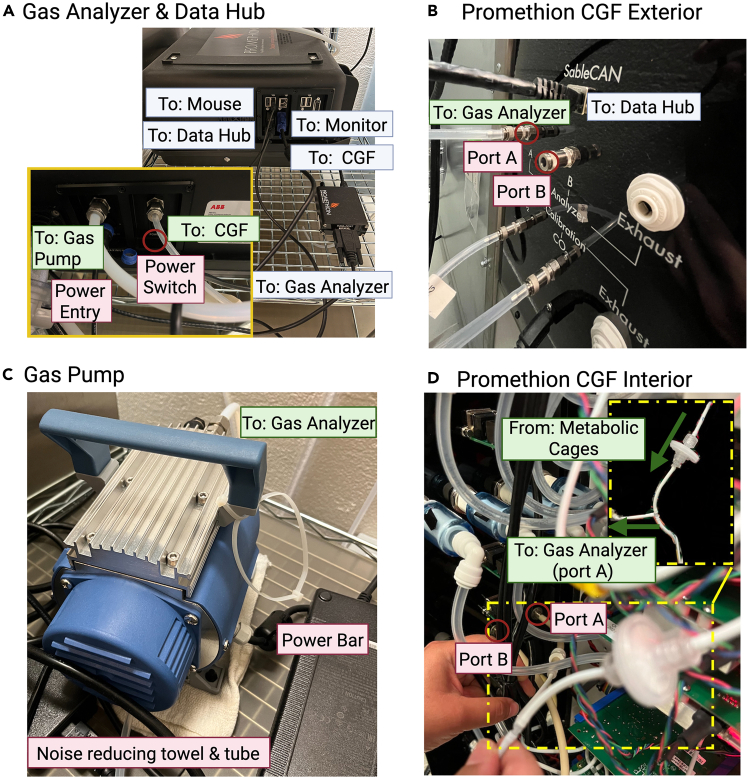
Gas analyzer, CGF, and pump connections (A) Gas analyzer and data hub connections. (B) Promethion CGF connections. (C) Gas pump connections. (D) Tubing connection inside the CGF that directs some of air flow from the cages to analyzer port A or B.

2. Connect the monitor (via a VGA port), mouse (via USB port), and Promethion Data Hub to one side of the analyzer (Figure 2A).

***Note:*** The mouse and monitor are required to change settings and for diagnostics of the stable isotope gas analyzer.

3. Connect the other end of Promethion Data Hub to a CAN port on the Promethion CGF analyzer using a standard RJ45 cable (Figure 2B).

***Note:*** No dedicated power input is required for the Promethion Data Hub.

4. Connect the analyzer and power cord to the pump (Figure 2C).

**CRITICAL:** The pump must be powered off when the isotope analyzer is not in use, as the closed flow paths can cause damage if the pump remains on.

***Note:*** If the pump vibration generates excessive noise that interferes with mouse experiments, connect a tube to the bottom of the pump (see Figure 2C). Placing a cloth beneath the pump can further reduce noise.

5. Connect the power cord to the analyzer and use a tube to connect the analyzer with the Promethion CGF analyzer (Figures 2A and 2B).
6. Open the Promethion CGF analyzer and ensure the gas from the cages diverts to the analyzer port (A or B) connected with gas analyzer (Figure 2D).

**Figure 3:**
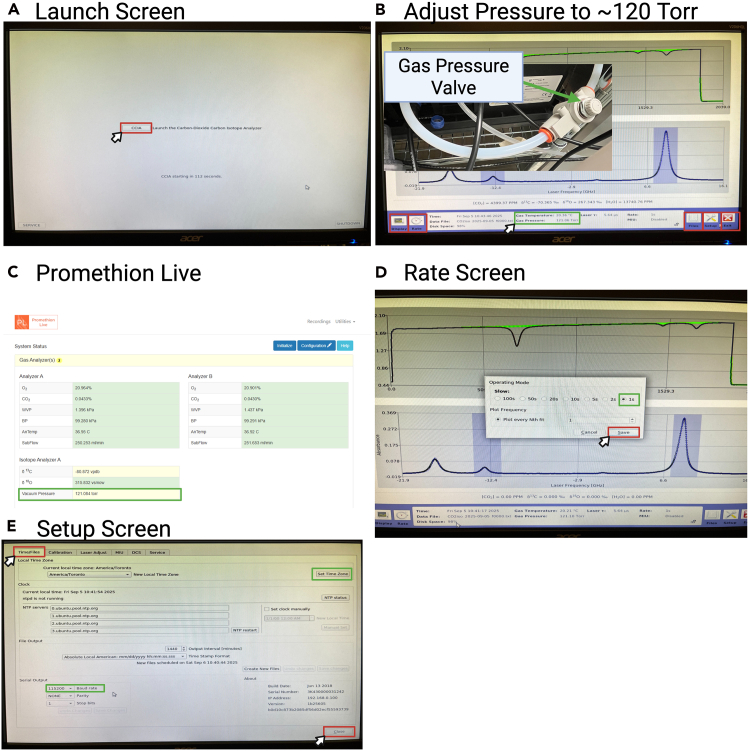
Stable Isotope Gas Analyzer initialization and setup (A) Launch screen. (B) Adjust pressure to ∼120 Torr. (C) Pump pressure ∼120 Torr shown on Promethion Live. (D) Rate screen. (E) Setup screen.

### Initializing and setting up the stable isotope gas analyzer

**Timing: 15 min**

7. Switch on the power button on the side of the analyzer (see in Figure 2A). The launch service screen will display (see Figure 3A).
8. Click on the launch button (CCIA) to launch the analyzer (see Figure 3A).

***Note:*** The analyzer will automatically launch itself if no selection is made within 120 s.

9. Turn on the vacuum pump and adjust the vacuum pressure to ∼120 Torr (see Figure 3B).

***Note:*** When the isotope analyzer is connected to the CGF, the Promethion Live will automatically detect the analyzer and the vacuum pressure will be visible (see Figure 3C).

10. Click on the Display tab at the bottom left of the screen to select one of the 4 display screens (numeric display, alarm status display, spectrum display, time chart display).
11. Click on the Rate tab at the bottom left of the screen to select the rate at which the data is recorded (see Figure 3D).

***Note:*** The default frequency for data recording (1 s) is recommended, because this is the same frequency as the Promethion measurements.

12. Click on the Setup tab on the bottom right of the screen and then select the Time/Files tab to set the baud rate and time zone (see Figure 3E).

***Note:*** The baud rate should be set at 115200 for proper communication with the Promethion Data Hub.

**CRITICAL:** Do not adjust any settings in Calibration without consulting the expert personal and with proper training.

***Note:*** The Laser Adjust, MIU, DCS, and Service screens are not used.

13. Click the Close button to return to the home screens.
14. Click the Files tab at the bottom right of the screen to access the data.

***Note:*** Normally, the Promethion system intercepts and logs the data.

15. Click the Exit button and then Shutdown button to turn off the analyzer. Then shut off the vacuum pump and switch off the analyzer.

### Testing the stable isotope gas analyzer with 1-^13^C glucose in SPF mice

**Timing: 18 h**

This protocol describes detailed steps in validating the stable isotope gas analyzer using 1-^13^C glucose in SPF mice.

16. Turn on the stable isotope gas analyzer the night before the experiment.

**CRITICAL:** Accurate measurement requires thermal stabilization of the laser cavity in the stable isotope gas analyzer; a minimum of 2-h equilibration is necessary, with 16–24 h equilibration preferred.

***Optional:*** Record the ^13^CO_2_ baseline in the environment before mice acclimation. Ensure the Mass Monitors for body mass, water, and food of all cages are set to ‘None’ in the Promethion Live (cage settings) before recording.

17. Place mice in the Promethion cages (one mouse per cage, up to 8 cages) to acclimate for at least 2 h.
18. Record the ^13^CO_2_ baseline in the breath of each individual mouse during the 2-h acclimation period.

***Note:*** This baseline recording can be as little as 20 min.

19. Make 1-^13^C glucose solution in sterile saline with maximum gavage volume of 10 mL/kg.
20. Once acclimated, gavage mice with 1-^13^C glucose at different testing doses.

**CRITICAL:** Take note of the time immediately after each gavage. This information is needed during data analysis.

21. Stop recording after the ^13^CO_2_ value return to baseline.

***Note:***^13^CO_2_ peaks around 30 min after glucose gavage and returns to baseline in 2–2.5 h.

## Key resources table

**Table:** 

REAGENT or RESOURCE	SOURCE	IDENTIFIER
**Bacterial and virus strains**		

*Lactobacillus delbrueckii subsp. bulgaricus*	ATCC	11842

**Chemicals, peptides, and recombinant proteins**		

[3-^13^C] Sodium D-lactate	Cambridge Isotope Laboratories, Inc	CLM-10768-PK
[3-^13^C] Sodium L-lactate	Cambridge Isotope Laboratories, Inc	CLM-1579-0.1
D-Glucose (1-^13^C, 98-99%)	Cambridge Isotope Laboratories, Inc.	CLM-420-0.25
Lactulose (U-^13^C_12_, 99%)	Cambridge Isotope Laboratories, Inc.	CLM-9043-PK
[1-^13^C^fru^] lactulose	Omicron	LAC-009
Lactobacillus MRS Broth w/o Dextrose	Cepham Life Sciences	66416–0
Lactobacillus MRS Broth	MilliporeSigma	69966-500G
Difco Lactabacilli MRS Agar	Becton Dickinson	288210
Ultra LC/MS Grade Acetonitrile	Caledon Laboratories Ltd.	1405-7-40
Ultra LC/MS Grade Water	Caledon Laboratories Ltd.	8805-7-40
Ultra LC/MS Grade Methanol	Caledon Laboratories Ltd.	6705-7-40

**Critical commercial assays**		

PicoProbe D-Lactate Assay Kit (Fluorometric)	Abcam	ab174096
PicoProbe L-Lactate Assay Kit (Fluorometric)	Abcam	ab169557

**Experimental models: Organisms/strains**		

Mouse: C57BL/6N	Taconic Biosciences, Inc.	RRID:IMSR_TAC:B6
Mouse: C57BL/6N Germ-free	In-house colonies bred in the Farncombe Gnotobiotic Unit at the McMaster Animal Facilities	

**Software and algorithms**		

GraphPad Prism Version 10	GraphPad Software	RRID:SCR_002798

**Other**		

Promethion Metabolic and Behavioral Cages	Sable Systems International	
Stable Isotope Analyzer	Sable Systems International	
GasPak EZ Anaerobe Container System Sachets with Indicator	Becton Dickinson	260001
GasPak EZ Incubation Container, Standard, 15 Plates	Becton Dickinson	BD260671
MS Qu-TOF	Agilent	6546 LC/Q-TOF
Excella E24 Incubator Shaker	New Brunswick Scientific	
Lyophilizer	SP Scientific	BTP-9EL00W
Astec CHIROBIOTIC R Column	MilliporeSigma	13019AST
Free-Standing Microcentrifuge Tubes with Screw Caps	GeneBio Systems	MCS020

## Materials and equipment

• A single stable isotope gas analyzer can be configured to measure either cage numbers 1–8 or 9–16; switching between cage groups after setup is discouraged unless necessary.
• The stable isotope gas analyzer calibration settings (recommended by expert personal from Sable) are Total [CO_2_] ppm = 490.5; δ^13^C ‰ VPDB CO_2_= −8.0; δ^18^O ‰ VPDB CO_2_= −35.0. Calibrate the gas analyzer ONLY when you have odd values of Total [CO_2_] ppm, δ^13^C ‰ VPDB CO_2_, or δ^18^O ‰ VPDB CO_2_. Calibrating more often decreases the life of the unit. It is strongly advised not to change any of the settings in the calibration menu without external assistance.

**Table:** Metabolites extraction solvent

Reagent	Final concentration	Amount
Acetic Acid	5%	5 μL
Acetonitrile	85%	85 μL
H_2_O	10%	10 μL

Store at 25°C for 1 week.

## Step-by-step method details

### Bacterial culture with ^13^C-labeled lactulose

**Timing: 2–3 days**

This section describes the steps to culture *Lactobacillus delbrueckii subsp. bulgaricus (L. del)* with ^13^C-labeled Lactulose. This protocol can be adapted to other bacteria that produce the metabolites of interests.

1. Preparation for bacterial growth medium
  a. Prepare MRS medium. Mix the MRS (powder) with Milli-Q water based on the instruction of the manufacturer.
  b. Autoclave the MRS medium and allow medium to cool down to 25°C.
  c. Store the MRS medium at 4°C.
2. Rapid thawing of cryopreserved bacteria
  a. Retrieve the cryovial containing *L. del* from cyrostorage.
  b. Immediately immerse the cryovial in a 37°C bead or water bath.
3. Transfer bacteria to the culture flask.
  a. Pipet the bacteria suspension (approx. 1 mL) to the MRS medium (100 mL) in a 200 mL glass flask.
  b. Gently swirl the flask to evenly distribute bacteria in the medium.
  c. Place the culture flask in BD GasPak EZ container systems with anaerobic environment.
  d. After 30 min, check the anaerobic indicator attached to the BD GasPak EZ sachet (it should be white, not blue) to confirm that the anaerobic environment has been achieved.
  e. Incubate the bacteria at 37°C for 24 h in an incubator.
4. Count bacteria number.
  a. Prepare MRS agar plate. Mix the MRS agar (powder) with Milli-Q water based on the instruction of the manufacturer. Autoclave the mixture and allow to cool down to 40°C and pour 10–15 mL into each petri dish. Store the MRS agar plate at 4°C once it solidifies.
  b. Perform serial dilution (1:10^2^, 10^3^, 10^4^, 10^5^, 10^6^) of the bacteria culture in MRS medium from step 3 and plate the diluted bacteria (10 μL) on a MRS agar plate using an inoculating loop or a cell spreader.
  c. Place the MRS agar plate in the anaerobic BD GasPak™ EZ container systems for 24 h to allow colonies to grow.
  d. Count the number of visible colonies and calculate the bacteria number by multiply by the dilution factor to get the original bacterial concentration in colony-forming units per milliliter (CFU/mL).
5. Freeze down bacteria
  a. Prepare 30% glycerol with MRS medium and filter sterilize the solution with a 0.22 μm filter.
  b. Mix the bacterial culture (1:1, V/V) with the 30% glycerol, write the concentration (from step 4c) on the cryovial.
  c. Freeze the bacteria in −80°C freezer.
6. Prepare dextran-free MRS medium with 10 mg/mL 1-^13^C lactulose.
7. Culture 5 × 10^5^ CFU bacteria in 500 μL of MRS with C^13^-lactulose medium for 24 h in a 2 mL screw cap microcentrifuge tube.
8. Collect filter sterilized medium from three separate bacteria culture for ^13^C D-lactate and L-lactate quantification (see steps below).

**Pause point:** The filtered medium can be stored at −20°C for 1 month or at −80°C for long term storage.

### ^13^C D-lactate and L-lactate quantification in bacteria culture

**Timing: 2–3 days**

This section details the steps involved in quantifying D-lactate and L-lactate with assay kits and proportion of labeled and non-labeled D-lactate and L-lactate in bacteria culture medium using LC/MS. This protocol can be adapted to other microbial metabolites.

9. Determine the concentration of D-lactate and L-lactate in bacteria culture medium following the instructions of Abcam D- and L-lactate assay kits (D-lactate, ab174096; L-lactate, ab169557).
10. Extract metabolites in bacteria culture medium.
  a. Prepare metabolite extraction solvent (see formula in materials and equipment).
  b. Mix 1 part of sample with 4 parts of solvent (1:4 v/v), vortex and centrifuge at 10000 g for 10 min at 4°C.
  c. Transfer the supernatant to a new tube and lyophilize sample to concentrate metabolites.
  d. Reconstitute the samples with 25 μL of metabolite extraction solvent.

***Note:*** All reagents need to be LCMS grade (higher grade than HPLC).

**Pause point:** The reconstituted samples can be stored at 4°C for 1 week or −20°C for 1 month before the LC/MS analysis.

11. Perform LC/MS analysis on reconstituted samples using a chiral column.
  a. Prepare solvent A (15% v/v 33.3 mmol/L ammonium acetate and 85% v/v acetonitrile) and B (100% acetonitrile) and purge the Agilent Q-TOF to remove any air bubbles from the lines.
  b. Inject 20 μL of each sample for analysis. The same LC/MS settings were used in this paper.^4^
  c. Perform isotope analysis using the Batch Isotopeologue extraction Wizards in Agilent MassHunter Profinder (Ver. 10).
  d. On the Ion Species tab, select labeling ^13^C and put 99% as Isotope purity.
  e. Create a Personal Compound Database and Library (PCDL) with D-lactate and L-lactate, including their formula, mass and retention time.
  f. Select Finish, the software will automatically look for ^13^C labels in the targeted metabolites.

### Monocolonization of GF mice with a bacterial strain (*L. del*)

**Timing: 1 week**

This section describes the protocol for monoclonizing GF mice with D-lactate-producing bacteria (*L.del*). These mice will be used for ^13^CO_2_ measurement after ^13^C-labeled substrate administration. This monocolonization protocol can be adapted to other bacterial strains.

12. Prepare bacteria *L.del* and count the bacteria number as described in step 1–4.
13. Resuspend bacteria in 25°C sterile PBS (1 × 10^9^ CFU/mL).
14. Prepare negative pressure pathogen isolation cages.
  a. Autoclave all components of the isocages, including the beddings, nestlets, and water (bottles).
  b. Assemble the cages in a BSC.
15. Gavage 1 × 10^9^ CFU suspension (resuspended in a volume of sterile PBS equivalent to 10% of the body weight) into GF mice in a BSC to generate monocolonized mice.
16. House these monocolonized mice in the sterile isocages for one week to allow bacterial colonization.
17. Confirm bacterial colonization 7 days after bacteria gavage.
  a. Collect 2–3 fresh feces pallets into a pre-weighed 1.5 mL sterile Eppendorf tube.
  b. Weight the feces pallets and add 25°C sterile PBS (1:10, w/v) into the tube.
  c. Homogenize the feces in PBS using a bead beater.
  d. Centrifuge the tubes at 25°C at 3000 g for 5 min.
  e. Smear the supernatant onto a MRS agar plate.
  f. Place the MRS agar plate in the anaerobic BD GasPak EZ container systems for 24 h to allow colonies to grow.
  g. Visualize bacteria colonies to confirm the success of colonization.

***Optional:*** Perform 16S analysis to further confirm the identity of colonized bacteria, however, since MRS is selective for lactic acid bacteria, the presence of colonies will indicate that *Lactobacillus* colonization was successful.

### ^13^CO_2_ measurement in SPF, monocolonized, and GF mice after oral gavage of ^13^C-labeled substrates (D-lactate and lactulose)

**Timing: 3–8 h**

This section describes the protocol for measuring ^13^CO_2_ in the breath of SPF, monoclonized and GF mice, including germ-free cage preparation. This protocol can be adapted to other ^13^C- or ^18^O-labeled substrates. Please test the dose and the time course response of ^13^CO_2_ (make sure to see both peak and clearance) before the experiment.

18. Use a negative pressure pathogen isolation cage to house monoclonized and GF mice.
  a. Autoclave all components of the isocages, except for the mass monitors, the stoppers for the water bottle and the red acrylic windows of the body mass device.
  b. Assemble the cages and place GF mice in these cages in a BSC.

***Note:*** The mass monitors, the stoppers for the water bottle, and the red acrylic windows of the body mass device cannot be autoclaved but can be decontaminated/wiped down using Peroxiguard/Micro-Chem Plus.

19. Acclimate SPF, monocolonized and GF mice in the Promethion cages for 2 h and record baseline ^13^CO_2_ of each mouse during this period.
20. Gavage 3-^13^C sodium D-lactate (1.2 mg per mouse) to each mouse. Gavage GF mice first (in a BSC) and then monocolonized mice or SPF mice to minimize bacteria colonization in GF mice.
21. Stop recording after the ^13^CO_2_ value return to baseline.

***Note:***^13^CO_2_ peaks around 20–25 min after D-lactate gavage and returns to baseline in 2–2.5 h.

22. Let mice recover for 24 h and repeat step 19–21 with U-^13^C lactulose (1.2 mg per mouse).

***Note:***^13^CO_2_ peaks around 110–120 min after lactulose gavage and returns to baseline in 5–6 h.

### Quantification and statistical analysis of ^13^CO_2_

**Timing: 1–2 h**

This section details the step-by-step mathematical calculation of the substrate oxidization rate as well as statistical analysis and data presentation.

23. Download the recording data from Promethion Live once the recording is completed.
24. Use MacroInterpreter to analyze (standard analytical scripts) the raw data collected by Promethion Live and export the data as excel file (see sample data – a single mouse given 2.5 mg of 1-^13^C glucose).
25. Calculate the rate of ^13^C-labeled tracer oxidation.^3^
  a. The two data sets (see sample data) needed are VCO_2_ level (data in vco2 column) and the level of ^13^C enrichment in the exhaled CO_2_ during the length of recording (data in si13c column).
  b. Set the time of tracer administration as 0 min for each individual mouse.
  c. Calculate the average vco2 and si13c value one hour before tracer administration as baseline (0 min).
  d. Determine the time that it takes to oxidize the tracer. When the si13c values are comparable to the baseline at 0 min, this indicates that the tracer is completely oxidized by the animal.
  e. Convert si13c value to Atom Percent (AP) using the following equation:
    $$AP=\frac{100}{\frac{1}{\left(\left(\frac{si13c}{1000}\right)+1\right)*0.0112372}+1}$$
    ***Note:*** 0.0112372 is the Pee Dee Belemnite standard for carbon 13 to carbon 12 ratio.
  f. Calculate Atom Fraction Excess (AFE) using the following equation:
    $$AFE=\left(AP_{sample}-AP_{baseline}\right)/100$$
  g. Calculate tracer oxidation rate using the following equation:
    $$T=\frac{VCO_{2}*AFE}{q*k*m}$$
    ***Note:*** q is the number of ^13^C atoms in the tracer. k is the volume of CO_2_ produced per unit mass of tracer oxidized (mL/mg). The k value for some of the common tracers can be found in McCue et al.^5^ If the animal is burning a mixture of fuels, a value of 1 for k can be used.^6^ m is the molecular weight of the tracer.
  h. Calculate the cumulative tracer oxidation.
    ***Note:*** This value equals to the area under the curve of tracer oxidation rate.
26. Analyze the statistical significance of the cumulative tracer oxidation (e.g., t tests, One-way ANOVA based on the number of experimental groups).

## Expected outcomes

**Figure 4:**
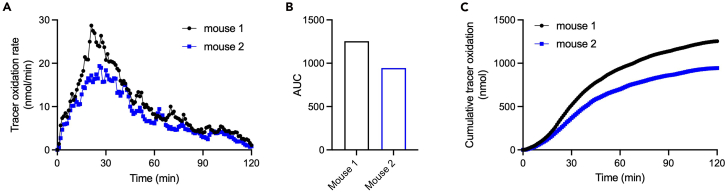
Example figures for expected outcomes (A) Tracer oxidation rate. (B) Area under the curve (AUC) of tracer oxidation rate. (C) Cumulative tracer oxidation.

The *in vitro* stable isotope ^13^C tracing in bacterial culture medium should show proportional incorporation of ^13^C in D-lactate. Ideally, little ^13^C incorporation is shown in other metabolites (e.g., L-lactate). After collecting the data from the stable isotope gas analyzer, the tracer oxidation rate (ug/min or nmol/min) of each animal is shown as a reverse U-shape figure (Figure 4A). The stable isotope tracer is administered orally at time 0. The critical metrics in this figure are magnitude and duration of the response and area under the curve (Figure 4B). Additionally, the cumulative stable isotope tracer oxidation can be calculated and presented (Figure 4C). Both GF and SPF mice should be able to metabolize D-lactate at the same rate. Since GF mice cannot process lactulose without gut bacteria, the lactulose oxidation rate should be close to zero. The lactulose oxidation rate in the monocolonized and SPF mice should be similar to what we see in Figure 4A, but compared with direct ^13^C D-lactate gavage, the peak of ^13^CO_2_ should appear much later considering the lactulose gut transit time and the time needed for gut bacteria processing lactulose into D-lactate.

## Limitations

The measurement of ^13^CO_2_ reflects whole-body oxidation and does not resolve tissue-specific contributions or alternative fates of the metabolite, such as incorporation into biosynthetic pathways. We also cannot rule out other possible sources of ^13^CO_2_. The approach is restricted to known microbial metabolites, as it requires prior identification of the substrate of interest. Furthermore, because the method relies on ^13^CO_2_ detection in exhaled breath, it applies only to substrates containing carbon or oxygen atoms that can ultimately oxidized to CO_2_. The protocol may be less reliable when metabolites are slowly absorbed, extensively metabolized in the liver before systemic circulation, and/or produced at levels too low for detection. In germ-free experiments, microbial contamination can confound results, making rigorous sterility essential.

## Troubleshooting

### Problem 1

Step 18–21, the peak response of ^13^CO_2_ (si13c value) in the breath is too low (200 or lower) to be distinguished from baseline noise.

### Potential solution

There are two ways of increasing the peak response of ^13^CO_2_ in the breath.

• Increase the dose of the substrate
• Increase the number of ^13^C-labeled carbons in the substrates.

### Problem 2

When recording the real-time ^13^CO_2_, one or multiple cages show low airflow rate consistently (highlighted in yellow in the Promethion Live). The optimal airflow rate for each cage is 2000 mL/min (highlighted in green in the Promethion Live). The low airflow rate could indicate a blockage in the tubing and may affect VCO_2_ measurements.

### Potential solution

Disconnect the tubing from the cage to run water through. This will clear the clog caused by beddings and debris in the cage. Let the tubing air dry before re-assemble to the cage.

### Problem 3

Bacteria did not grow well in dextran-free MRS medium supplemented with lactulose.

### Potential solution

• Test a few more strains of the bacteria that can produce the same metabolites.
• Switch to a different substrate (cannot be utilized by host) that may favor the growth of the bacteria.

### Problem 4

There is no change in the si13c values after administering the substrate.

### Potential solution

• Check the tubing connection inside the CGF (see Figure 2D), make sure there is a T tube that direct some of the air flow from the cages to the stable isotope gas analyzer.
• Inside the CGF, the tube should connect to the corresponding outlet (outside, analyzer A or B). Also ensure the mice are placed in the corresponding cages (analyzer A measures cages 1–8, analyzer B measures cages 9–16).

### Problem 5

Step 11, low incorporation of ^13^C label in bacterial metabolites.

### Potential solution

Increase labeled substrate concentration or incubation time or increase the number of ^13^C in the substrate.

## Resource availability

### Lead contact

Further information and requests for resources and reagents should be directed to and will be fulfilled by the lead contact, Dr. Jonathan D. Schertzer (schertze@mcmaster.ca).

### Technical contact

Technical questions on executing this protocol should be directed to and will be answered by the technical contact, Dr. Jonathan D. Schertzer (schertze@mcmaster.ca).

### Materials availability

This study did not generate any new unique reagents.

### Data and code availability

This study did not generate any new data or code.

## Acknowledgments

We thank the Center for Metabolism, Obesity, and Diabetes Research (MODR) for the Sable Promethion Core system and the Centre for Microbial Chemical Biology for support with LC-MS analysis. This work was supported by a 10.13039/100021557CIHR project grant (PJT-175054) and 10.13039/501100000196Canada Foundation for Innovation (CFI) to J.D.S. and MODR postdoctoral fellowship to H.F.

## Author contributions

H.F. performed all the experiments and analyzed the data. H.F., N.G.B., D.K.Z., R.R.e.-L., and J.D.S. wrote this protocol.

## Declaration of interests

The authors declare no competing interests.

## Supplemental information

Data S1. Substrate oxidation calculations, related to Step 24
